# No discrimination shock avoidance with sequential presentation of stimuli but shore crabs still reduce shock exposure

**DOI:** 10.1242/bio.019216

**Published:** 2016-06-15

**Authors:** Barry Magee, Robert W. Elwood

**Affiliations:** School of Biological Sciences, Queen's University, Belfast BT9 7BL, Northern Ireland, UK

**Keywords:** Avoidance, Discrimination, Electric shock, Nociception, Pain, Shore crab

## Abstract

Insights into the potential for pain may be obtained from examination of behavioural responses to noxious stimuli. In particular, prolonged responses coupled with long-term motivational change and avoidance learning cannot be explained by nociceptive reflex but are consistent with the idea of pain. Here, we placed shore crabs alternately in two halves of a test area divided by an opaque partition. Each area had a dark shelter and in one repeated small electric shocks were delivered in an experimental but not in a control group. Crabs showed no specific avoidance of the shock shelter either during these trials or in a subsequent test in which both were offered simultaneously; however they often emerged from the shock shelter during a trial and thus avoided further shock. More crabs emerged in later trials and took less time to emerge than in early trials. Thus, despite the lack of discrimination learning between the two shelters they used other tactics to markedly reduce the amount of shock received. We note that a previous experiment using simultaneous presentation of two shelters demonstrated rapid discrimination and avoidance learning but the paradigm of sequential presentation appears to prevent this. Nevertheless, the data show clearly that the shock is aversive and tactics, other than discrimination learning, are used to avoid it. Thus, the behaviour is only partially consistent with the idea of pain.

## Abstract (precis)

**Summary:** There was no discrimination shock avoidance with sequential presentation of stimuli but, consistent with pain, shore crabs used other tactics to reduce shock exposure.

## INTRODUCTION

Animal pain is difficult to investigate because a behavioural response to a noxious stimulus might be a nociceptive reflex (Sherrington, 1906) that lacks an associated unpleasant experience (Crook and Walters, 2011; Elwood et al., 2009; Elwood, 2011) and because of the difficulty in gaining access to the feelings of animals (Stamp Dawkins, 2012). For humans, pain is defined as “an unpleasant sensory and emotional experience associated with actual or potential tissue damage, or described in terms of such damage” (IASP, 1979); however this definition is not suitable for animals because they cannot describe their experiences. Various definitions have been proposed for pain in animals such as “an aversive sensory experience caused by actual or potential injury that elicits protective and vegetative reactions, results in learned behaviour, and may modify species specific behaviour” (Zimmermann, 1986). Sneddon (2009) refines this definition suggesting that animals in pain should “quickly learn to avoid the noxious stimulus and demonstrate sustained changes in behaviour that have a protective function to reduce further injury and pain, prevent the injury from recurring, and promote healing and recovery.”

These definitions rely largely on behavioural and physiological indicators (reviewed by Sneddon et al., 2014) that have enabled recent investigations into the possibility of pain in birds (Gentle, 2011) and fish (Sneddon, 2009; Braithwaite, 2010). The evidence for these vertebrates is consistent with the idea of pain and suffering. Various experiments on decapod crustaceans and cephalopod molluscs have also produced results consistent with the idea of pain. For example, complex prolonged grooming and rubbing of an antenna was seen in glass prawns (*Palaemon elegans*) that had been subject to noxious chemical treatment with the behaviour directed at the specific antenna, but was reduced if treated with a local anaesthetic (Barr et al., 2008). Hermit crabs subjected to abdominal electric shock within their shell were more likely to evacuate from a less-preferred species (Elwood and Appel, 2009) or evacuate from a less-preferred species at a lower voltage (Appel and Elwood, 2009a). That is, they showed a trade-off between shock avoidance and retention of valuable resources. Further, shocked hermit crabs that did not evacuate were more likely to approach and move into a new shell and did so more quickly than non-shocked crabs (Elwood and Appel, 2009), a shift in motivation that lasted at least 24 h (Appel and Elwood, 2009b). Crayfish exposed to aversive electric fields showed anxiety-like behaviour coupled with physiological changes (Fossat et al., 2015), and crabs exposed to shock showed physiological signs of stress (Elwood and Adams, 2015). Cephalopods showed long-term motivational change after receiving a wound in squid (Crook et al., 2011) and octopus (Alupay et al., 2014), including long-term directed wound attention in the later.

These observations in invertebrates are not consistent with a reflex response and thus the idea of pain in decapods and cephalopods cannot be dismissed as mere nociception (Elwood, 2011) but we accept that neither can any of these studies be taken as proof of pain (Elwood, 2012). We do, however, note that the idea of pain is often accepted for vertebrates but dismissed for invertebrates that show similar responses (Sherwin, 2001). The evidence for pain in invertebrates and fish has been dismissed because they do not have morphologically identical brain structures to those implicated in human pain (Rose et al., 2014). This argument against pain, however, is logically flawed because entirely different central nervous systems may have the same function albeit with very different structure. For example, both decapods and cephalopods have excellent visual abilities despite lacking a visual cortex similar to that in humans (Elwood, 2011). This reluctance to consider evidence for pain in invertebrates in an impartial manner reflects a general lack of concern for the well-being of invertebrates (Horvath et al., 2013). Given the vast numbers of decapods used in the food industry, without regard to potential suffering, it is vital that we ask if these animals might have the potential to feel pain (Elwood, 2012).

The key function of pain is to enable swift avoidance learning and discrimination learning to gain long-term protection from further tissue damage (Bateson, 1991; Broom, 2001; Sneddon, 2009; Crook et al., 2014). Such learning was shown in fish (Dunlop et al., 2006) and in shore crabs (*Carcinus maenas*) (Magee and Elwood, 2013). In the latter study crabs were given ten trials in a brightly lit arena in which there were two dark shelters. Crabs received a shock on entry to one shelter and at 5 s intervals whilst they remained in that shelter. If a crab emerged from the shelter the shock ceased but if it went to the alternative shelter no shock was given. Each trial lasted 2 min after which the crab was removed from the arena, then replaced in the centre and allowed to make another choice. On the third trial more crabs changed to the alternative shelter if they had received shock in the previous trial, thus showing swift avoidance and discrimination learning. Some crabs stopped entering shelters over the ten trials but for those that did enter, a significantly higher number used the non-shock shelter. A final test determined what had been used to discriminate between the shelters. Distinctive patterns above the shelters were switched for some crabs but this had no effect on the choice. Further, during the first ten trials the crab was always oriented the same way but in the discrimination trial some were turned 180°. These crabs now were more likely to move to the previous shock shelter indicting that discrimination had been on the basis of walking either to the crab's left or right. That is, the crabs showed response learning rather than place learning (e.g. de Bruin et al., 1997). That result was unexpected given previous work showing an ability of crabs to associate visual stimuli with a negative stimulus (e.g. Tomsic et al., 2003).

Whilst discrimination learning was demonstrated by Magee and Elwood (2013), the number of visits to the shock and non-shock shelters varied between subjects because they could choose which to enter. Indeed, a few crabs failed to visit the shock shelter during the ten trials. Nevertheless, the use of two shelters was an improvement on previous experiments that employed just one location in which a shock was given and an increased latency to enter the shock area taken to indicate avoidance learning (Denti et al., 1988). That result might not indicate learning because it could be due to a general decreased ability or motivation to walk rather than an association between walking to a location and shock. By giving a choice of shelters Magee and Elwood (2013) demonstrated response learning resulting in choice of a safe location rather than shifts in latency of movement; however, that paradigm also allowed the animals to leave the shock shelter and enter the non-shock shelter within a single trial. Whilst this might be similar to situations on the shore, where numerous shelters may be available, it resulted in different crabs having different experiences of the two shelters during training.

Here, we overcame this difficulty by placing shore crabs in a brightly lit arena with two shelters separated by an opaque partition so that only one shelter was available per test, thus all crabs had broadly similar experience during training. As in Magee and Elwood (2013) the crab orientation was kept the same during ten training trials in which the crab was alternately placed on either side of the partition. One side, randomly selected, was the shock side and the other the non-shock side so the subjects were equally exposed to the shock and non-shock shelter. To determine if discrimination learning occurred after the ten training trials we tested without the partition but with all other stimuli the same. This was followed by a second test in which crab orientation and visual stimuli were varied to determine which aspects may have been learned (Magee and Elwood, 2013). Thus, during training the stimuli were presented sequentially whereas they were simultaneously presented in Magee and Elwood (2013). We note, however, that sequential paradigms have been shown to result in inferior learning in other discrimination learning studies (e.g. Dyer and Neumeyer, 2005).

If crabs experience pain and show discrimination learning during the training trials we predicted that fewer crabs would enter the shock shelter than the non-shock shelter, particularly in later trials. Further, although these animals normally require a dark shelter (Barr and Elwood, 2011), they might hesitate if that shelter is associated with shock. Thus we predicted that crabs in a shock shelter trial would take longer to enter than those in a non-shock shelter trial. We also predicted that crabs would be more likely to leave the shock shelter, particularly in later trials and leave more quickly in later trials if they learned that leaving terminates shock. Finally, although we sought to avoid autotomy of appendages, we predicted that if it were to occur it would be during shock rather than non-shock trials. In the discrimination tests we predicted that more crabs would enter the non-shock shelter than the shock shelter when given a choice in the first test without the partition. If these animals showed a preference for the non-shock shelter we predicted that, as in a previous experiment (Magee and Elwood, 2013), they would use direction of movement rather than visual cues to discriminate between shelters. Although in the design noted above there was an alternation of shock and non-shock trials we also employed a separate control group that had identical treatment except that no shock was delivered in any trial.

## RESULTS

The first trial of the experimental group was with the shock shelter and all 76 crabs entered on that trial, however fewer crabs entered that same shelter in subsequent trials (Table 1) (χ^2^_4_=16.63, *P*=0.002). For trials with the non-shock shelter there was no significant variation between trials (Table 2) (χ^2^_4_=3.09, *P*=0.54). Importantly, there was no difference in the probability of entry to the shock and non-shock shelters on each of the five pairs of trials (Fisher exact tests all *P*>0.1). Further, there was no difference between the experimental and control groups in the number of crabs entering the shelter in any of the ten trials (Fisher exact tests all *P*>0.1). There was no difference between the two groups in the number of crabs that entered on all occasions or failed to enter a shelter on at least one occasion (15/28 vs 40/76, Fisher *P*=1.00).

**Table 1.**
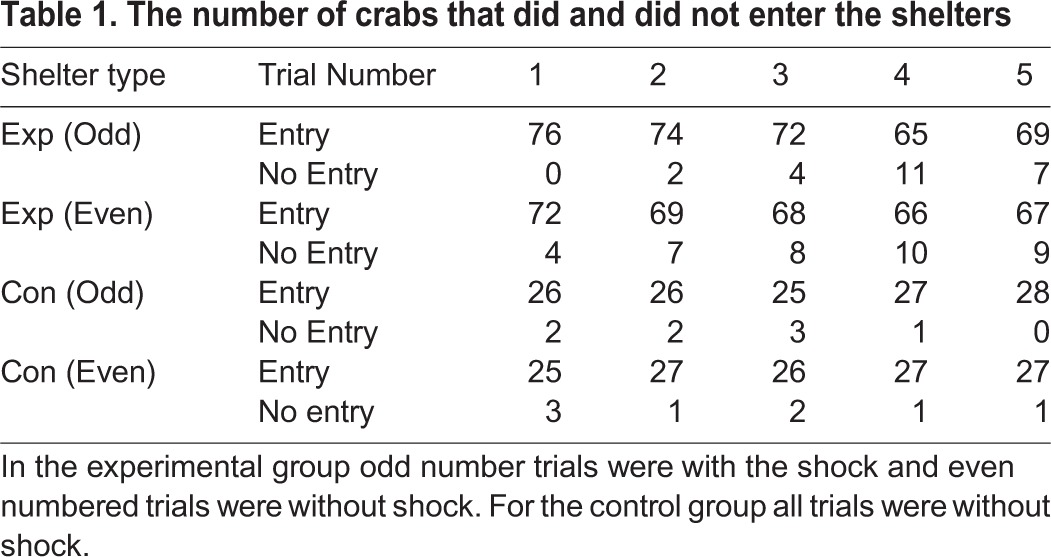
**The number of crabs that did and did not enter the shelters**

**Table 2.**
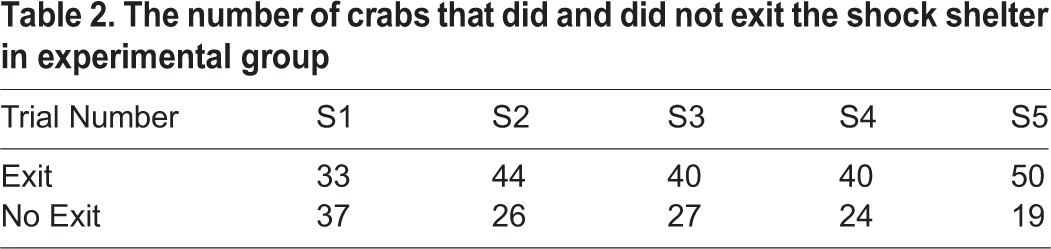
**The number of crabs that did and did not exit the shock shelter in experimental group**

**Fig. 1.**
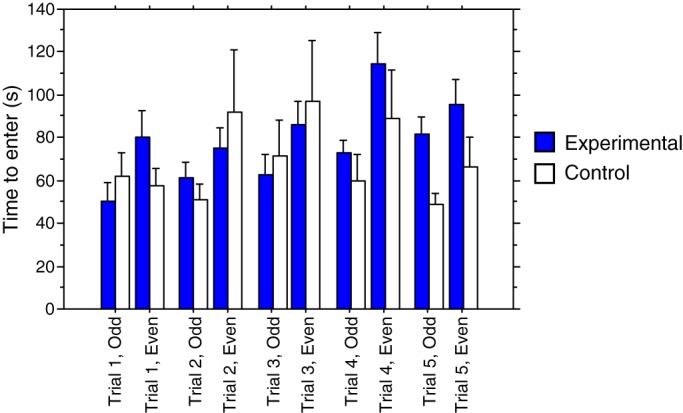
**Mean time taken to enter the shelters in seconds (±s.e.m.) for each trial as used in a repeated measures ANOVA.** Note that for the experimental group ‘odd’ trials were with shock whereas ‘even’ trials were without shock. With the control group there was no shock at any time. For the experimental group *n*=76 and for the controls *n*=28, however, some animals did not enter the shelter on some tests. Full details of number of animals entering on each trial is in Table 1.

To investigate latency to enter the shelters we examined those 40 experimental and 15 control crabs that entered the shelter on all ten trials using repeated measures ANOVA. Unexpectedly, the latency to enter a shelter in the even numbered trials (non-shock in both groups) was greater than in the odd-numbered trials (shock in the experimental group) (*F*_1,212_=12.1, *P=*0.001; Fig. 1), but there was no change over the course of the trials (*F*_4,212_=1.99, *P=*0.097) or between the experimental and control groups (*F*_1,212_=0.66, *P*=0.42) and none of the interaction effects were significant (all *P*>0.1).

Some crabs in the experimental group that entered the shock shelter subsequently left that shelter on being shocked (Table 2), and the probability of these exits increased over the five shock trials (G^2^_4_=9.69, *P*=0.0459) (note that crabs that autotomised during a trial were excluded from this analysis because autotomy disrupted the application of the shock on that trial). Crabs were considerably more likely to exit a shock shelter than a non-shock shelter (Fisher's exact test, shock/non-shock pairs 1-5, *P*<0.0001 for all pairs of trials), as no crab left a non-shock shelter. No crab in the control group left a shelter after entry on any trial.

**Fig. 2.**
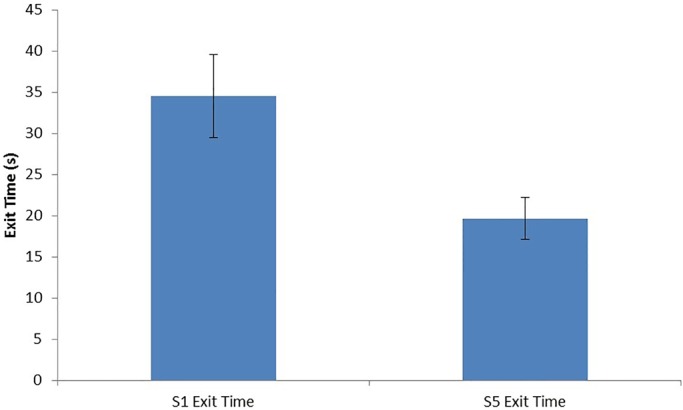
**Mean times in seconds (±s.e.m.) taken to exit for those animals in the experimental group that left the shock shelter on both the first and fifth trial with the shock shelter (*n*=29) (paired *t*-test).** In the experimental group 29 crabs exited the shelter in both the first and last trial with the shock shelter. The latency (seconds ±s.e.m.) prior to exit was significantly greater in the first compared to the last trial (paired *t*-test).

Shock was terminated if the crab left the shock shelter and those that exited on both the first and last trial of the experimental group were compared, which showed that latency to exit markedly decreased with experience (Paired *t*-test, t_28_=3.31, *P=*0.0026; Fig. 2).

Significantly more crabs autotomised in the experimental than in the control group (21/89 vs 0/28 Fisher test *P*=0.0033). Crabs in the experimental group only autotomised in a shock shelter (binomial test, 21:0, *P*<0.001).

When the central partition was removed for the experimental group 66/76 entered a shelter but there was no preference for the former non-shock side (*n*=36) compared to the former shock side (*n*=30) (goodness of fit, χ^2^=0.55, *P*=0.46). There was no difference in the latency to enter the former non-shock and shock shelters (unpaired *t*-test, non-shock: 122.3±10.6 s, shock: 114.9 s, t_64_=0.47, *P*=0.64). Thirty crabs moved to their left to access the chosen shelter whereas 36 moved to their right (goodness of fit, χ^2^=0.55) and 27 chose vertical stripes whereas 39 chose horizontal stripes (χ^2^=2.18, *P*=0.14).

In the second test of the experimental group without the partition, 61 crabs entered a shelter. Again there was no preference shown for the former non-shock shelter (*n*=29) over the former shock shelter (*n*=32) (goodness of fit, χ^2^=0.15, *P*=0.7). The latency to enter the former non-shock shelter did not differ from the former shock shelter (unpaired *t*-test, non-shock: 104.0±8.7 s, shock: 95.7±8.9 s, t_59_=0.67, *P=*0.51).

For the experimental group, 59 crabs entered a shelter in both tests after partition removal. In the second test, some crabs were tested without any change whereas others had some conditions altered. There was no significant difference in latency to enter across the four sets of conditions (ANOVA, *F*_3,55_=1.14, *P*=0.34). Some crabs went to the same shelter in both tests whereas some changed their choice; however, the number that changed varied according to the condition (Table 3; χ^2^=10.11, *P*=0.0176). Most crabs returned to the same shelter if there was no change in condition (12/13) and fewer went back to the same shelter if both orientation and background were changed (6/17) (Fisher exact test *P*=0.0024), but there was no difference between those that just had the background or just the orientation changed (Fisher test, *P*>0.99). When data were grouped, those that had the same visual stimulus were more likely to go to the same shelter than were those with the visual stimuli switched (Fisher 19/24 vs 17/35, *P*=0.029). When the data were grouped with respect to crab orientation those with the same orientation were more likely to go to the same shelter than were those with their orientation switched (Fisher *P=*0.036). When grouped to determine if the crabs went in the same direction relative to their body then 37 went in the same direction and 21 in a different direction (binomial *P=*0.048). When the data were grouped to determine if crabs went to the same stripe or the different stripe configuration in the second test, 36 went to the same and 22 to the different configuration (binomial *P=*0.087).

**Table 3.**
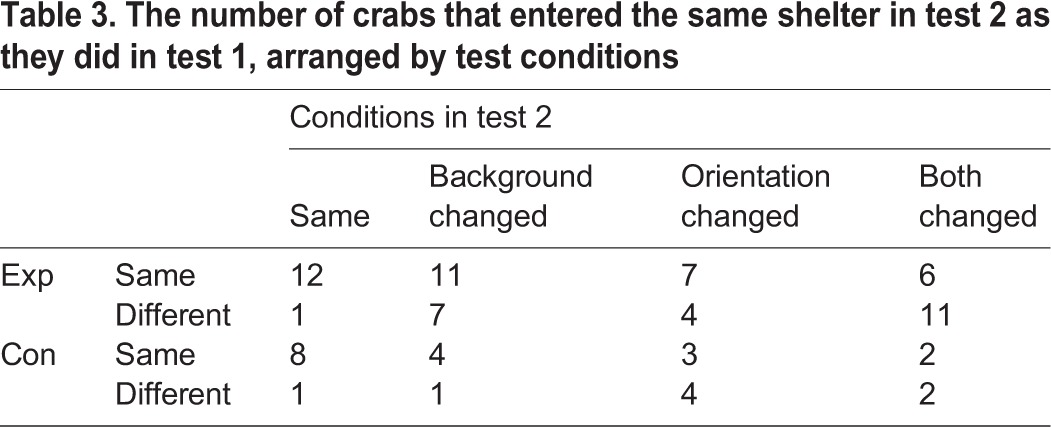
**The number of crabs that entered the same shelter in test 2 as they did in test 1, arranged by test conditions**

When the partition was removed for the control group and the crabs had a free choice, 25 entered a shelter with 12 moving to the left and 13 to the right. On the second test after partition removal 27 entered a shelter with 15 moving to the left and 12 to the right. The sample size was too small to test for overall differences in choice depending on the final conditions. When data were grouped, of those tested with the same position of the stripes 11/16 went to the same side as in the first test, whereas of those with the stripes switched 6/9 went to the same side (Fisher *P*=1.0). When grouped with respect to crab orientation, those with the same orientation as in the first test tended to go to the same side whereas those that had their orientation changed also tended to change their choice (12/17 vs 2/8 Fisher *P*=0.08). Overall crabs moved in the same direction relative to their body in the second test as in the first test (18 vs 7 binomial *P*=0.042) but they did not go to the same type of stripe (14 vs 11 binomial *P*=0.69).

When data for the two groups were combined more crabs moved in the same direction relative to their body in the second test than moved in the opposite direction (binomial 55 vs 28 *P*=0.004) but they did not go to the same type of stripe (binomial 50 vs 33 *P=*0.078).

## DISCUSSION

Pain enhances the salience of a noxious stimulus and thus functions to facilitate swift avoidance learning (Bateson, 1991; Elwood, 2011). Previous experiments have been consistent with the idea of pain in decapods (Barr et al., 2008; Appel and Elwood, 2009a,b; Elwood and Appel, 2009) and, in particular, demonstrated rapid discrimination between shelters and avoidance of the one associated with the aversive experience of an electric shock (Magee and Elwood, 2013). Thus, in the present study we predicted that fewer crabs would enter the shock shelter than the non-shock shelter, particularly during the later stages of the training period of ten trials; however, this did not occur.

Further, there was no difference between experimental and control groups in shelter entry for any trial. Neither was there a significant difference between the experimental and control in the number of crabs entering a shelter on any trial. That is, receiving shock on alternate trials did not change the response of these animals with respect to shelter entry. Thus, it is clear that there is no indication of discrimination and avoidance of the shock shelter during training.

When the partition was removed the crabs were given a choice of shelters but again there was no evidence of discrimination and avoidance of the shock shelter for the experimental group.

We had predicted that if discrimination learning occurred then crabs would be hesitant about going to the shock shelter during the training trials and thus would take longer than those going to the non-shock shelter. Further, this difference should be more marked in the final training trials. This should not occur in the control group, thus ANOVA should show an interaction between treatment group and trial number and possibly an overall increase in latency in the experimental treatment; however, these predictions were not upheld from this experiment. There was no main effect of treatment on the latency to enter shelters, no overall effect of trial number and no interaction term. Surprisingly, there was an overall effect of odd and even trials (odd trials in the experimental group were the shock trials); however, the greater latencies were in the even trials which were non-shock trials in the experimental group. Further, separate ANOVAs (not presented) showed a significant effect in both groups so this alternation of short and long latencies could not have been due to the shock in the experimental group. We currently have no explanation of why crabs should show this pattern for latency to enter shelters. The conclusions from the results on probability of shelter entry and of latency to enter the shelter do not match our predictions and do not support the idea of pain. They are markedly different from those found in a previous study (Magee and Elwood, 2013).

Three major differences between Magee and Elwood (2013) and the present study might account for the very different results. First, many studies on discrimination learning have noted that simultaneous presentation of the two stimuli produces swifter learning than sequential presentation (e.g. Dyer and Neumeyer, 2005). Further, in mate choice experiments the better quality mate could also not be determined with sequential presentation compared to simultaneous presentation, suggesting a perceptual problem with the former design (Dougherty and Shuker, 2015). Second, when both shelters were simultaneously presented (Magee and Elwood, 2013) some crabs left the shock shelter and went to the non-shock shelter within the same trial; however, in the present study, whilst crabs could leave the shock shelter the barrier prevented them from reaching the alternative shelter. Being able to move between shelters within a single trial might have facilitated learning in Magee and Elwood (2013) because of the very short interval between sampling the shock and then the non-shock shelter. Third, the configuration of the test arena remained much the same throughout the experiment of Magee and Elwood (2013) but in the present study each crab was placed alternately in separate halves of the arena and was then placed into a new situation with the entire area available in the final tests for discrimination learning. These changes might have inhibited the discrimination learning because crabs were unable to determine the spatial relationships between the two shelters. We cannot be sure which of these possibilities might have separately or in combination produced the major differences in learning between Magee and Elwood (2013) and the present study; however, a variety of studies have shown that different learning paradigms often result in different learning abilities in crustaceans (Denti et al., 1988; Fernandez-Duque et al., 1992). Indeed, just changing the position of the animal has a marked influence on avoidance learning in crayfish (Kawai et al., 2004).

Although the crabs clearly failed to discriminate between the shelters they employed other tactics to avoid or reduce the number of shocks. In the field these crabs are found under rocks during day light and thus avoid predation (Barr and Elwood, 2011). That virtually all crabs entered a shelter on the first training trial indicates the high motivation of these animals to avoid bright light and find shelter; however, an increasing proportion of subjects exited the shock shelter during the trial sequence. By contrast, no crab left the non-shock shelter and no crabs left shelters in the control group, thus, crabs might learn that leaving the shelter stops the shock. Indeed, this idea is supported because crabs in the experimental group showed a significant decline between the first and fifth trials in the time taken to get out and avoid further shock; however, for this tactic to be used the crabs must overcome their motivation to avoid bright light. A dark shelter is a valuable resource (Fathala and Maldonado, 2011; Magee and Elwood, 2013) and moving out into the light area indicates that the shock is an aversive stimulus because the crabs give up the resource to avoid shock. Giving up a valuable resource to avoid shock has been considered to be consistent with pain in vertebrates (Millsopp and Laming, 2008) and invertebrates (Barr and Elwood, 2011; Elwood and Appel, 2009). The present data, however, would also be consistent with the idea of sensitization to the noxious stimulus as occurs in cephalopods (Crook et al., 2011, 2013; Alupay et al., 2014). Thus, the increased probability of moving out of the shelter, coupled with decreased latency, might be due to the greater aversion to the stimulation rather than learning per se. However, sensitization could equally enhance learning but which possibility holds cannot be determined with the present data.

The aversive nature of shock is further indicated because no crab in the experimental group autotomised an appendage during a non-shock trial, but approximately 26% autotomised an appendage during a shock trial and the appendage was always one to which shock was delivered. Further, no crab in the control group autotomised. Autotomy is commonly seen in crabs and it involves muscular contraction causing the limb to break along a proximal plane without haemolymph loss (Patterson et al., 2007). Loss of one leg appears to have a minimal effect on walking ability (Magee and Elwood, 2013).

As noted above, when both shelters were first offered simultaneously, the experimental crabs did not base their decision on whether they had experienced shock in one of those shelters. There was no significant bias to using the non-shock shelter and there was no difference in the latency to enter the shock or non-shock shelter, thus, no discrimination learning was shown immediately after the ten training trials. However, in the second discrimination test, the choice of shelter was not random and instead there was a preference for a particular set of cues that were consistent with those used in the first simultaneous test. When both treatment groups were analysed, with respect of whether they went to the same type of stripe, there was no significant bias for either group or for the two groups combined. By contrast, when each group was analysed to see if they went in the same direction with respect to their body (i.e. if they walked to their left or right) then both groups showed a significant bias for moving in the same direction as in the first test. The most parsimonious explanation is that crabs might have individual bias in walking direction (i.e. to their left or right). In this case discrimination learning need not be invoked. However, that could not account for the finding in Magee and Elwood (2013) that showed clear evidence of discrimination learning between previous shock and non-shock shelters by response learning rather than by visual discrimination. In that previous study the final preference for a direction of movement was determined by shock experience in a particular shelter.

To conclude, there is no evidence for discrimination learning during the sequential presentation of shelters resulting in shock or not. We note that discrimination learning is typically difficult with sequential presentation of stimuli compared to simultaneous presentation (Dyer and Neumeyer, 2005), which seems to apply to shore crabs (Magee and Elwood, 2013); however, despite being unable to discriminate between the two shelters during the course of the ten sequential training trials, crabs used other tactics to markedly reduce the number of shocks. In particular, there was an increase in the proportion of crabs that left the shock shelter over the five trials with that shelter and those that left the shock shelter did so more quickly in later trials. These observations on exiting shelters in which shock is delivered are consistent with expectation of pain (Zimmermann, 1986; Sneddon, 2009; Sneddon et al., 2014). Future studies on potential pain avoidance by discrimination leaning should take into account limitations of perceptual abilities when choices are offered sequentially rather than simultaneously.

## MATERIALS AND METHODS

The key collection, housing and much of the experimental methods are the same as Magee and Elwood (2013) but this is a separate experiment with different subjects, thus there is no overlap in data. Subjects were European shore crabs, *Carcinus maenas*, collected from Barr Hall Bay in Strangford Lough, Co Down, Northern Ireland, using baited pots. Fully intact crabs with carapace width of 5-8 cm were placed in plastic holding tanks (76×38×17 cm) and transported to Queen's University, Belfast within 6 h, with a maximum of 50 crabs per tank. Seaweed (*Ascophyllum nodosum*) was also taken from the site for shelter and to reduce agonistic interactions within the tanks. The crabs were housed in the same tanks with seaweed, filled with aerated seawater to a depth of 5 cm and fed with Tetra Pond Floating Food Pellets (Melle, Germany). They were maintained in a cold room at 11-13°C with a 12 h light/dark regime, with the water changed every 3 days and the food added immediately after.

Crabs (*N*=80) were tested individually in a glass tank (62×25×25 cm) with dark shelters, made from dark sheet plastic positioned at opposite ends of the tank (each 11×25 cm), leaving an open area (40×25 cm) between the shelters. We randomly (dice) positioned two distinctive patterned cards to cover the end walls of the tank above the shelters. One pattern consisted of vertical and the other horizontal black and white stripes of equal width (1.8 cm) and total area. A removable opaque central partition was then placed into the tank to provide two equal sections each with one shelter, with an open area (20×25 cm) between each shelter and the central partition. Gravel was placed on the floor of the tank and seawater added to a depth of 1 cm before the tank was placed into an observation chamber (71×36×39 cm) behind a one way mirror. An energy saving bulb, equivalent to 100 W (3430 lux, measured by a Precision Gold N76CC Light Meter, Yorba Linda, CA, USA), was suspended over each section of the tank. Each crab had a loop of insulated copper wire (0.2 mm diameter) placed around each of the fifth walking legs, with the other end of each wire attached to a Grass S9 electric stimulator (West Warwick, RI, USA), the insulation was removed at both ends of the wire. The left and right legs had wires that were randomly attached to the positive and negative terminals of the stimulator, which was set to deliver an electric shock of 10 V at 180 Hz for 200 ms. The crab was placed into a randomly (dice) selected side of the tank facing towards the observer behind the one-way mirror, this first side of the tank was used as the shock side throughout the initial phase of the experiment whereby the subject was shocked if it entered the shelter.

If the crab entered the shelter it received an electric shock as soon as its entire carapace was under the shelter and received another shock every 5 s for 2 min or until it exited the shelter. If the crab did not exit within the 2 min it was then removed from the shelter. If the crab exited the shelter it remained inside the tank for 2 min before being removed, if during this time it re-entered the shelter it was shocked again. If the crab did not enter a shelter during the trial within 10 min it was removed from the tank.

In the next trial the crab was placed into the other section of the tank, again facing the observer. This section contained the other background card and was the non-shock side of the experiment for the initial training phase. If the crab entered the shelter there was no shock delivered and the crab was allowed to remain there for 2 min before removal. If the crab did not enter the shelter within 10 min it was removed from the tank.

Between each trial the crab was placed for 2 min in an adjacent seawater-filled container with a loosely fitted lid. The room was dark for this period except for the last 10 s, when the lid was removed and the energy saving bulbs turned on, to allow them to attain full brightness. This process was repeated a further four times, allowing the crab a total of five experiences in each section of the tank, alternating between the two sections.

Following this phase the central partition was removed to enable access to both shelters. The background cards remained in the same positions. Two minutes after the last training trial the crab was again placed into the tank facing the observer. If the crab entered either shelter it was not shocked and was allowed to remain in the shelter for 2 min before being removed. The shelter that the crab entered was recorded with reference to previous shock or non-shock experience. If the crab did not enter a shelter within 5 min it was removed from the tank. This test determined if the crab could associate one shelter with the shock and avoid it.

A second test then determined which aspects of the situation might have been learned. To assess if the visual stimulus (horizontal or vertical stripes) was involved some crabs had the stimuli in the same locations whereas for others they were switched. To assess if the direction of movement was involved some crabs were placed again facing the observer whereas others were placed facing away from the observer. Thus, for example, if previously they had walked to the left to the non-shock shelter, now they would have to walk to the right. These two types of conditions formed a sub-experiment in a 2×2 design. Again if the crab entered either shelter it was not shocked. The shelter that the crab entered was recorded with reference to previous shock or non-shock location. If the crab did not enter a shelter within 5 min it was removed from the tank. Upon removal after the second test each crab was sexed and carapace width was measured.

We monitored the crabs for autotomy, a defensive reaction by which an appendage is cast off at a specific breakage plane, leaving a sealed limited wound (Patterson et al., 2007). If autotomy occurred, the connection with the wire was lost so the wire was attached to the adjacent walking leg in preparation for the next trial. Of the 80 crabs tested, 22 autotomised a leg. One crab autotomised during handling but the remaining 21 autotomised a rear walking leg within a shelter. Four of these crabs autotomised a second appendage and were removed from subsequent analysis because of potential impaired movement. Loss of a single appendage did not impair movement and did not affect latency to enter either the shock shelter of the non-shock shelter on the fifth trials with those shelters (unpaired *t*-tests: fifth shock trial, autotomy: 70.8±9.0 s, no autotomy: 80.1±8.6 s, t_66_=−0.63, *P*=0.53; fifth non-shock trial, autotomy: 114.9±24.3 s, no autotomy: 113.6±15.9 s, t_65_=−0.26, *P*=0.80) (note not all crabs entered on the fifth trials in either shelter).

A control group of crabs was treated in an identical manner except that no shock was delivered on any trials (*n*=28). Thus the treatment of the two groups differ on the odd numbered trials (shock or not) but not on the even numbered trials.

No licence is required for this work in the United Kingdom because crabs are presumed not to experience pain. Nevertheless we attempted to follow guidelines of the Association for the Study of Animal Behaviour by keeping the shock level to a minimum (determined from previous work) and keeping the sample size as low as possible. Thus we elected to use the minimum suitable for our eight groups for the final test with categorical data being used. Further, we anticipated that some crabs would drop out of the data set and indeed four did so because two legs autotomised. Autotomy is common in the wild and about 10% of our original samples had one or more limbs missing without apparent harm. Crabs were in the laboratory for a maximum of two weeks and then returned to Strangford Lough. We have no reason to believe that subsequent survival of the animals was compromised. We further minimized the numbers used by electing not to have additional experimental groups. We considered additional procedures including no shock followed by shock and random allocation to shock and no shock sides of the apparatus but concluded that the likely gain in information would have been small in comparison to the large numbers of crabs being exposed to shock.

Data will be provided on request to the corresponding author.
